# Asymmetric LAMP–gold nanoparticle biosensing for rapid detection of Kenyan tomato leaf curl virus isolates from crude extracts

**DOI:** 10.1039/d6ra04196e

**Published:** 2026-05-28

**Authors:** Abigarl Ndudzo, Florence Ng'ong'a, Edith K. Avedi, Elijah M. Ateka

**Affiliations:** a Department of Molecular Biology and Biotechnology, Pan African University Institute of Basic Sciences, Technology and Innovation, JKUAT Kenya; b Department of Biochemistry, Jomo Kenyatta University of Agriculture and Technology Kenya; c Phytosanitary and Biosecurity Department, Kenya Plant Health Inspectorate Services Kenya; d Office of the Dean, School of Agriculture and Environmental Sciences, Jomo Kenyatta University of Agriculture and Technology Kenya; e Department of Biotechnology, Faculty of Environmental and Life Sciences, Lupane State University Zimbabwe andudzo@lsu.ac.zw

## Abstract

Tomato leaf curl virus (ToLCV) is a significant constraint to tomato production, particularly in resource-limited agricultural settings where access to sensitive, decentralized diagnostic tools is limited. While PCR-based detection methods require specialized equipment and exhibit reduced sensitivity when applied to crude field samples, many isothermal methods generate double stranded amplicons that are suboptimal for downstream signal transduction. To address these issues, an asymmetric loop-mediated isothermal amplification (ASYLAMP) strategy was developed to preferentially generate single-stranded amplicons compatible with gold nanoparticle (AuNP) hybridization, enabling rapid visual detection. The optimized ASYLAMP assay achieved shorter time-to-positive results compared to symmetric LAMP while maintaining equivalent specificity, as supported by melt curve analysis and statistical comparison. The assay exhibited a limit of detection of 0.0008 fg µL^−1^ and showed no cross-reactivity with other tomato-infecting viruses. Successful AuNP functionalization and target hybridization were confirmed by characteristic plasmonic shifts in UV-visible spectra. Evaluation of 79 field samples using crude DNA extracts demonstrated that the ASYLAMP–AuNP biosensor detected more positives than PCR, with all biosensor-positive samples independently confirmed by real-time qPCR. The biosensor demonstrated high diagnostic sensitivity and specificity, substantial agreement with the gold standard (*κ* = 0.92), and strong discriminatory performance (binary ROC AUC = 0.96), supporting its reliability as a qualitative diagnostic tool. This study introduces a proof-of-concept chemically integrated ASYLAMP–AuNP biosensor combining high sensitivity, specificity, and analytical performance. By aligning amplification chemistry with plasmonic signal transduction, the platform offers a potential diagnostic solution for plant virus surveillance in resource-constrained settings.

## Abstract 2

Asymmetric LAMP generates more ssDNA amplicons, followed by efficient hybridization with DNA probe, yielding stable AuNP-complementary DNA association for a solution-based visual readout.

## Graphical abstract

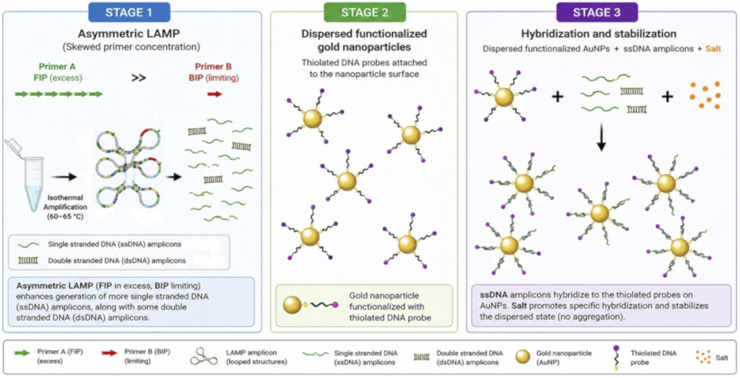

## 1 Introduction

Plant viral pathogens are a major cause of crop losses worldwide, reducing yields and quality and seriously impacting food security and local economies.^1^ Traditionally, the main defense against viruses has been the plant's own immune system; however, when this is insufficient, preventing infection becomes the only alternative. This makes early diagnosis pivotal for controlling and eliminating plant viruses. An important example is the tomato leaf curl viruses, a significant group of single-stranded DNA viruses in the genus *Begomovirus*, family *Geminiviridae*, which are spread by the whitefly *Bemisia tabaci*.^1^ These viruses cause tomato leaf-curl complexes and related problems that can sharply reduce yields. In Kenya, early studies identified recombinant begomoviruses in tomato crops, including tomato leaf curl Uganda virus.^2^ Metagenomic studies later revealed that tomato leaf curl Arusha virus, likely from Tanzania, is the primary begomovirus causing leaf curl symptoms in Kenya.^3^ Surveys further revealed disease prevalence ranging from 19.5% to 64% in key tomato-growing areas, with higher rates in fields using conventional or recycled seed.^4^ More recent work by Kimathi *et al.*^5^ further expanded the understanding of begomoviruses in the region. Developing and adopting better diagnostics and integrated disease management are essential to minimize crop losses and protect farmers, especially as begomoviruses become more diverse and prevalent. Critically, tomato leaf curl disease can reduce yields by up to 100% when plants are infected early, further threatening food security and the livelihoods of smallholder farmers in Africa and other tropical and subtropical regions.^3^ To address this, fast and accurate detection of ToLCV is essential for monitoring, using clean seeds, informing vector control decisions, and setting up quarantine measures. Without early and rapid diagnosis, outbreaks can spread unnoticed through planting material and whitefly populations.^6^ The situation in Africa is especially serious. Several tomato-infecting begomoviruses co-circulate; mixed infections are common; and shifts in whitefly populations and their range, partly due to climate change, have made these diseases even more widespread, underscoring the need for easy-to-use diagnostic tools in the field.^3^

ToLCVs are of significance because they spread quickly through the highly mobile whitefly vector, often mix and recombine with local virus strains and satellite DNAs to create new, more harmful or adaptable strains,^2,3,5^ and affect many staple or cash crops that smallholder farmers rely on for their livelihoods and nutrition.^6^ In tomatoes and other valuable vegetables, early infection causes stunted growth and significant yield and market-quality losses, since rapid laboratory diagnosis is mostly centralized and inaccessible when needed. This puts smallholder incomes and household food security at risk across Africa and Asia.^7^ As a result, surveillance and seed health checks are often ineffective, allowing the virus to spread quietly through symptomless plants, traded seedlings, and whitefly vectors.^8^ In Africa, new or locally adapted ToLCV variants have been found in countries such as Tanzania, Kenya, Mali, and Burkina Faso. The region faces both ongoing and emerging ToLCV threats, exacerbated by climate change, expanding whitefly populations, and weak seed systems.^2,3^ These epidemiological factors mean that surveillance and early diagnosis are not just academic concerns; they are essential for sanitation (roguing), seed health certification, the use of resistant varieties, and targeted vector control to help prevent regional outbreaks and protect smallholder farmers.^7^

Several methods are available for ToLCV detection, including serological tests, the polymerase chain reaction (PCR), isothermal amplification, and nanobiosensors. Serological assays and lateral flow tests are simple, low-cost, and provide quick on-site results. However, they need specific, high-quality antibodies, which may not be available for all ToLCV variants. In addition, these tests are less sensitive to early or low-level infections and can cross-react, limiting their use for early detection or in genetically diverse ToLCV populations.^9^ On the other hand, PCR and qPCR are the laboratory gold standards for specificity and sensitivity. Many published assays target ToLCV rep gene (AC1), coat protein gene (cp), or intergenic regions, and can detect very low virus titres depending on the primers and protocols used.^10,11^ However, PCR and qPCR require specialized equipment, cold storage for reagents, and trained staff, making them difficult and expensive to use in the field, especially for smallholder farmers in Africa.^12^ While PCR remains the gold standard, it is not practical for field use. In response, isothermal amplification methods with lateral flow or colorimetric readouts show promise for quick, field-ready detection.^13^ New portable diagnostic tools, such as loop-mediated isothermal amplification (LAMP) and nanoparticle-based biosensors, are promising for ToLCV detection in the field. They are more sensitive and rapid compared to conventional PCR methods.^12,14^

Isothermal amplification methods such as LAMP and recombinase polymerase amplification (RPA) allow nucleic acid amplification at a single moderate temperature, eliminating the need for thermocyclers and simplifying the required equipment.^15^ Many LAMP assays have been published.^12,16,17^ LAMP is quick, taking 10 to 45 minutes, and is compatible with crude extracts. However, it has some drawbacks, including a high risk of contamination, which can lead to false positives; ambiguous colorimetric readouts in complex plant extracts; and lower sequence-level specificity when results are based solely on total DNA detected.^18^ Highly sensitive and specific diagnostic tools can rapidly identify viral infections, guide effective control measures, and limit their spread. To overcome the identified limitations of LAMP, several new nanoparticle-based approaches have been developed.^19,20^ These nanoscale materials have unique properties that have enabled important advances. For example, gold nanoparticles (AuNPs) are commonly used in biosensing because they can produce colorimetric or plasmonic signals upon binding to specific DNA sequences or upon aggregation in response to a target. To achieve greater specificity, AuNPs are functionalized with ligands (to form Au nanoprobes) that bind only to the analyte of interest, such as a thiolated oligonucleotide matching the target sequence.^21^ Furthermore, when combined with hybridization or target-growth chemistry, AuNP probes can achieve high analytical sensitivity.^22,23^ Several strategies are used in AuNP-based colorimetric assays, including cross-linking and non-cross-linking methods. In this study, a non-crosslinking approach was chosen, in which a single-stranded DNA probe adsorbs onto the citrate-stabilized AuNP surface. Upon hybridization with its complementary DNA target, a duplex forms, increasing negative charge density and steric stabilization around the AuNPs. This enhanced stabilization strengthens electrostatic repulsion, preventing aggregation upon salt addition and allowing the red color to persist.^24,25^ If no complementary DNA is present, probes do not hybridize, leaving AuNPs less stable. Salt induces aggregation, causing a visible color change from red to blue or purple due to plasmon coupling.

Several studies on AuNP virus diagnostics report detection limits for various viruses^24–27^ often in the femto- to pico-molar range, though results can vary depending on the platform. This shows the strong potential of AuNP readouts for sensitive, instrument-free diagnosis. Studies also note that crude plant extracts can affect assay reliability unless the chemistry and probe design are optimized.^26,28^ Phurijaruyangkun *et al.*^29^ reported a clinical LAMP–AuNP workflow for *Treponema pallidum*, in which LAMP amplified the target DNA, and an AuNP probe provided a clear colorimetric signal. The detection limit for both LAMP and LAMP–AuNP was 11.6 pg µL^−1^ in that matrix, and the method showed considerable specificity and was suitable for field use. These studies show that combining amplification with sequence-specific AuNP probes can provide both sensitivity and sequence discrimination, but the detection limits depend on the assay format, target, sample matrix, and probe chemistry.

Asymmetric LAMP and similar isothermal methods provide a useful means of linking isothermal amplification to simple AuNP readouts.^15^ This approach adjusts primer compositions or utilizes modified primers to create more single-stranded or probe-accessible loop regions, which helps advance specific probe binding for colorimetric, nanoparticle, or lateral-flow detection.^26,30^ In this study, an asymmetric LAMP (ASYLAMP) strategy was used by adjusting primer concentrations to favor the production of single-stranded DNA amplicons during amplification. This approach was designed to improve accessibility of the amplified target for hybridization with the AuNP probe, thereby enhancing hybridization efficiency, colorimetric signal clarity, and overall specificity of the AuNP-assisted detection system. Variants such as five-primer or stem-loop (ASLAMP) designs have yielded improved sequence-validated results and made lateral-flow interpretation clearer in other pathogen tests, while still working well at 60–65 °C. Pairing asymmetric LAMP with AuNPs provides a practical balance: high sensitivity and sequence specificity, with minimal equipment required and easy-to-read results for field use.^31^ These features make asymmetric LAMP a strong choice when a simple, equipment-free AuNP test is needed to distinguish similar viral strains in crude plant extracts.^30–32^ The skewing of primer stoichiometry avoids the need for any post-amplification processing for the generation of single-stranded DNA, thus presenting an assay that can easily find application at the point of need.

Although LAMP and AuNP probes each have their own strengths, a combined, standardized LAMP + AuNP platform for detecting Kenyan ToLCV isolates remains lacking. Early and highly sensitive detection is important for epidemiology because ToLCV can spread even with low vector numbers and often causes asymptomatic infections at first. Infected planting material and symptomless seedlings are key means by which the disease spreads.^3,7^ However, tests that detect the virus only after symptoms appear or at high copy numbers are not sufficient for seed certification, quarantine, or sanitation. Notably, LAMP assays can detect as few as hundreds of copies^12^ or even femtogram-level DNA in the lab. Similarly, AuNP probes alone can reach attogram-level sensitivity^27^ or detect single copies when used with amplification.^26^ By combining these methods, it may be possible to detect low levels of the virus in crude extracts early in infection and obtain a sequence-specific visual result that helps avoid false positives.

Africa faces several challenges, including a lack of centralized diagnostic facilities, limited cold storage capacity, insufficient laboratory staffing, and the need to process large numbers of samples for seed systems. Because of this, point-of-need diagnostics should be fast, compatible with crude extracts, and require minimal equipment.^12^ LAMP works with simple sample preparations and can use basic heaters, while AuNP readouts show color changes visible to the eye. A well-designed LAMP–AuNP workflow that uses crude buffer sampling, as seen in field LAMP studies, and gives a stable color result,^25,33–35^ could help meet this important need.

Genetic diversity and mixed infections in Africa make it challenging to achieve diagnostic specificity. ToLCVs readily recombine and form complexes with satellites, so assays relying solely on broad amplification may misidentify infections or fail to detect new variants.^36^ Adding a sequence-specific AuNP probe targeting conserved coat protein/replication protein motifs, while accommodating minor single-nucleotide polymorphisms, to a LAMP workflow enhances specificity. The LAMP reaction amplifies low amounts of template, while the AuNP probe specifically hybridizes to the amplified product, confirming that the amplified DNA contains the expected motif sequence. This step avoids false positives from non-specific LAMP products and allows the assay to distinguish between different ToLCVs.^25,37^ Given the high diversity of ToLCV isolates in Africa, diagnostic assays must incorporate local sequence differences. If primers or probes fail to match local variants, false negatives can occur.^34,38^ Studies show that local sequence variation can cause mismatches, so it is important to design and test diagnostics using region-specific data and local viral diversity.^39^

To support surveillance, seed certification, and rapid response, it is important to have validated limits of detection (LOD) and proven field performance in relevant African samples. However, there is no unified, systematically validated LAMP–AuNP assay for Kenyan ToLCV isolates that is benchmarked against conventional PCR, tested in crude extracts from key African tomato and chili pepper samples, validated across local ToLCV variants, and supported by clear limit-of-detection metrics and robustness testing. Filling this gap would help improve the timeliness of disease control in Africa and provide a practical tool for extension services, seed companies, and regulatory agencies. LAMP allows rapid, field-ready amplification, and AuNP probes add sequence specificity without specialized instruments. By demonstrating reliable detection limits, specificity, and performance with crude samples, the resulting test could rapidly enhance surveillance and disease management.

## 2 Methods

### 2.1 Sample collection and total DNA extraction

A field survey was conducted from September to November 2024 to collect leaf samples (Research license: NACOSTI/P/24/33998) from Embu, Kirinyaga, Narok, Laikipia, Baringo, and Kajiado counties in Kenya. In total, 79 leaf samples were collected. Young, topmost leaves were gathered from tomato and chili pepper plants. Both symptomatic (showing chlorosis/yellowing of young leaves, reduced leaf size, upward curling of margins, stunting, and flower drop) and asymptomatic plants were included. Samples were transported to the Pan-African University Institute of Basic Sciences, Technology and Innovation, Molecular Biology laboratory for analysis. DNA was extracted from ∼100 mg of leaf tissue using the GeneAll® Exgene™ Plant SV kit, following the manufacturer's instructions. DNA concentration was measured using a NanoDrop spectrophotometer (Genova Nano, Jenway), and integrity was assessed on a 1% agarose gel.

### 2.2 Conventional PCR assay for detection of Kenyan ToLCV isolates

The purified DNA (Section 2.1) was used as a PCR template. PCR was performed using the ToLCVF3 and ToLCVB3 primers designed using the PrimerExplorer v5 software (https://primerexplorer.jp/e/) targeting the AV1 coat protein gene of Kenyan ToLCV isolates (as described in Ndudzo *et al.*^40^). The reaction was carried out in a total volume of 25 µL containing 12.5 µL of Q5 2× Master Mix (New England Biolabs, US), 0.5 µL each of the ToLCVF3 and ToLCVB3 primers, 2 µL of template DNA (∼80 ng µL^−1^), with the volume made up with nuclease-free water. The mixture was incubated in a thermal cycler (ProFlex PCR Thermocycler, Applied Biosystems, USA) at 98 °C for 30 s for the first denaturation step, followed by 35 cycles at 98 °C for 10 seconds (s), 60 °C for 45 s, and 72 °C for 30 s, and the final extension was done at 72 °C for 5 minutes. The PCR amplicons were analyzed by 1% agarose gel electrophoresis in 1× TAE buffer at 100 mV for 1 hour, and the gel was visualized using the VILBER E-BOX CX5.TS EDGE (France). The *pMG-Amp* plasmid carrying the AV1 coat protein gene was used as the positive control, healthy plant DNA as the negative control (indicating the absence of the target sequence in uninfected plant samples), and nuclease-free water as the no-template control (to monitor contamination).

### 2.3 Conventional LAMP assay for detection of Kenyan ToLCV isolates

LAMP primer design targeted the AV1 coat protein gene, and the consensus sequence was derived from a multiple sequence alignment of Kenyan ToLCV isolates (MN894493.1, MN894495.1, MN894499.1, MN8944501.1, MN894503.1, and MN894504.1). The primers were designed using PrimerExplorer v5 software (https://primerexplorer.jp/e/) (as described by Ndudzo *et al.*^40^) and synthesized by GenScript, Netherlands (Europe). The LAMP assay was performed using the LAMP WarmStart® Master Mix kit (New England Biolabs, US) according to the protocol's guidelines. For the initial LAMP reaction, DNA from confirmed positive ToLCV samples was used. Briefly, each 25 µL reaction contained 12.5 µL of the master mix, 1.6 µM each of the forward inner primer (FIP) and backward inner primer (BIP), 0.8 µM each of the forward loop primer (LF) and backward loop primer (LB), 0.2 µM each of the forward primer (F3) and the backward primer (B3), 2 µL of DNA template (∼80 ng µL^−1^) and the volume was made up to 25 µL using nuclease free water. The *pMG-Amp* plasmid was used as the positive control, healthy plant DNA as the negative control, and nuclease-free water as the no-template control. The reaction mixture was incubated at 65 °C for 30 minutes in a water bath for the colorimetric assay, and color changes were observed. Approximately 5 µL of the LAMP product was analysed using 1% agarose gel. The real-time LAMP assay was conducted using the qTower384 thermal cycler (Analytik Jena, Endress + Hauser Company). The thermal cycling protocol included amplification at 65 °C for 30 s per cycle for 60 cycles, followed by inactivation at 95 °C for 2 minutes. Melt curve analysis was performed by increasing the temperature from 60 °C to 95 °C. The cycle threshold (*C*_t_) was used to determine the time to positivity (*t*_p_). The threshold time was defined as the cycle number at which fluorescence reached the relative fluorescence units (RFU) threshold. Each cycle lasted 30 seconds. *t*_p_ was calculated using the following formula:
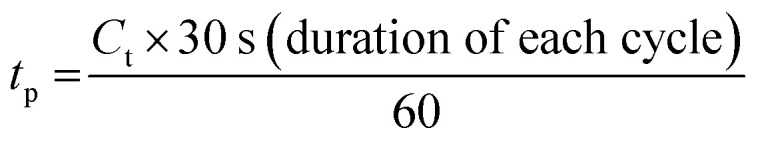


All reactions were carried out in triplicate.

### 2.4 Optimization of asymmetric LAMP for the detection of Kenyan ToLCV isolates

Asymmetric LAMP (ASYLAMP) increases the production of single-stranded loop regions or strand displacement products, making them easier for hybridization probes to detect and improving hybridization on the gold nanoparticle. This effect was achieved by introducing an imbalance in primer stoichiometry. For optimizing the asymmetric LAMP reaction, DNA from confirmed ToLCV-positive samples was used. ASYLAMP was performed in a 25 µL reaction volume containing 12.5 µL of WarmStart® 2× LAMP Master Mix (New England Biolabs, US).

Outer primers (F3 and B3) were each used at 0.2 µM, and loop primers (LF and LB) at 0.4 µM. To favor single-stranded product formation, the inner primers were added at different concentrations: the forward inner primer (FIP) at 2.0 µM (excess primer) and the backward inner primer (BIP) at 0.4 µM (limiting primer). Then, 2 µL of the DNA template (∼80 ng µL^−1^) was added, and nuclease-free water was used to bring the final volume to 25 µL. The *pMG-Amp* plasmid served as the positive control, healthy plant DNA as the negative control, and nuclease-free water as the no-template control. To optimize the reaction, BIP concentration was kept constant while FIP was systematically increased (stepwise) and compared to symmetric LAMP. To enhance the generation of single-stranded DNA (ssDNA) complementary to the AuNP-conjugated detection probe, asymmetric LAMP was performed by increasing the concentration of the FIP primer relative to BIP. FIP was specifically selected for excess optimization because the detection probe was designed complementary to the FIP-derived amplification strand. Colorimetric detection was performed in a water bath at 65 °C for 30 minutes. Real-time LAMP detection and *t*_p_ calculations were performed as described in Section 2.3. The asymmetric LAMP approach was tested using FIP : BIP ratios of 3 : 1, 5 : 1, and 8 : 1 (Table 1).

**Table 1:** Asymmetric LAMP optimization matrix^a^

Condition	FIP : BIP	FIP (µM)	BIP (µM)	FIP vol (40 µM)	BIP vol (µM)
Symmetric control	1 : 1	1.6	1.6	1.00 µL	2.00 µL
Asymmetric 1	3 : 1	1.2	0.4	0.75 µL	0.50 µL
Asymmetric 2	5 : 1	2.0	0.4	1.25 µL	0.50 µL
Asymmetric 3	8 : 1	3.2	0.4	2.00 µL	0.50 µL

a All reactions were run in triplicate.

### 2.5 Determination of analytical sensitivity and specificity of ASYLAMP

The analytical sensitivity of the asymmetric LAMP assay was determined using 10-fold dilutions (10^−1^ to 10^−12^) of total DNA from a confirmed ToLCV positive sample (∼80 ng µL^−1^). Approximately 2 µL of each dilution served as a template for both conventional PCR and asymmetric LAMP assays, following the methods in Sections 2.3 and 2.4. The detection limit was defined as the lowest DNA concentration yielding a positive result. Specificity was tested using DNA from ToLCV-infected samples and cDNA from other tomato-infecting viruses, including tobacco mosaic virus (TMV), cucumber mosaic virus (CMV), tomato mosaic virus (ToMV), impatiens necrotic spot virus (INSV), tomato spotted wilt virus (TSWV), and potato virus Y (PVY). The *pMG-Amp* plasmid served as a positive control, total DNA from a healthy tomato as a negative control, and nuclease-free water as a no-template control.

### 2.6 Data analysis

The collected real-time data were analyzed using the qTower384v1.0 software (AnalytikJena, Endress + Hauser Company), and GraphPad Prism v10.6.1 was used for statistical analysis. All experiments were performed in triplicate, and quantitative data are presented as mean ± standard deviation (SD) unless otherwise stated. Time-to-positive (*t*_p_) values obtained from symmetric and asymmetric LAMP reactions were compared to evaluate differences in amplification kinetics. Because symmetric and asymmetric LAMP assays were prepared as independent amplification reactions with distinct primer stoichiometries and reaction assemblies, *t*_p_ values were treated as independent experimental measurements. Accordingly, statistical comparisons between amplification formats were performed using an unpaired two-tailed *t*-test. Statistical significance was defined as *P* < 0.05. Exact *P* values were calculated and reported for all comparisons. All negative control (NC) and no-template control (NTC) reactions were excluded from statistical comparisons due to the absence of detectable amplification. Statistical analyses and graphical representations were generated using GraphPad Prism v10.6.1 (GraphPad Software Inc., San Diego, CA, USA).

### 2.7 Preparation and characterization of gold nanoparticle–DNA conjugates

Gold nanoparticles (AuNPs) with an approximate size of 20 nm were purchased from BBI Solutions (UK). The AuNP–DNA conjugate was prepared using the salt-aging method described by Jauset-Rubio *et al.*,^41^ with minor modifications. The thiolated DNA probe, manually designed to target the sequence between the Fc3 and Bc3 of the designated primer set (as described in Ndudzo *et al.*^40^), was synthesized by GenScript (Netherlands, Europe). To activate the probe, 100 µL of 100 µM thiolated DNA was mixed with 1 µL of 10 mM tris(2-carboxyethyl)phosphine (TCEP) and 2 µL of 500 mM acetate buffer at pH 5.2, then incubated for 1 hour at room temperature with gentle shaking. The tubes were covered with aluminium foil. TCEP was used to break the disulfide bond between the thiol and sulfhydryl groups in the DNA. Next, 1 mL of the AuNP suspension was added, and the mixture was gently mixed by inverting the 1.5 mL microcentrifuge tubes. The tubes were wrapped in aluminium foil and incubated overnight (16 hours) in the dark with gentle shaking. After this, 100 µL of 1 M NaCl and 10 µL of 500 mM Tris-acetate buffer at pH 8.2 were slowly added to the AuNP–DNA mixture, with 10 µL added every 20 minutes and gentle mixing after each addition. Salt aging was performed to increase the aptamer loading density while preventing AuNP aggregation. After this step, the mixture was incubated in the dark at room temperature for 24 hours with gentle shaking. Subsequently, bovine serum albumin (BSA) was added to a final concentration of 1% (w/v) (10 mg mL^−1^ in the AuNP–DNA mixture), and the mixture was incubated for 1 hour to block unmodified sites and reduce nonspecific adsorption to the AuNP surface. The AuNP–DNA conjugate was centrifuged at 15 000 rpm, 10 °C for 30 minutes. The pellet was then washed twice with 500 µL conjugate buffer (5 mM sodium borate, pH 8.8, with 1% w/v BSA and 10% w/v sucrose) to remove excess thiol-labeled DNA, and re-suspended in 50 µL conjugate buffer. The functionalized AuNPs were characterized using a Cary 100 Bio UV-Visible Spectrophotometer (Agilent) over wavelength range 400–800 nm. Data were analyzed and plotted using OriginPro 2025b. The final conjugate was stored at 4 °C in the dark until use.

### 2.8 Optimization of ASYLAMP – AuNP reaction for detection of the Kenyan ToLCV isolates

After the ASYLAMP assay was conducted, the AUNP-labeled DNA thiol probe was added to the LAMP products and hybridized at 65 °C for 5 minutes in the presence of various concentrations of MgSO_4_. To establish the optimal salt concentration for inducing free probe aggregation, the ASYLAMP product was hybridized to AuNP-labeled DNA probe under various concentrations of MgSO_4_, including 20, 40, 80, and 100 mM. The ratio of the AuNPs-labeled DNA thiol probe to ASYLAMP product to MgSO4 was maintained at 1 : 1 : 1 (5 µL each, giving a total volume of 15 µL). The hybridization reaction was observed with the naked eye, and color changes indicating positive (red) and negative (purplish-blue/blue-gray) reactions were noted and recorded. The solution color was confirmed by UV-Vis spectrophotometry. To optimize the hybridization temperature for LAMP product detection, 5 µL of AuNPs probe solution was added to the LAMP products at 59 °C, 62 °C, 65 °C, and 68 °C for 5 minutes each.

### 2.9 Sensitivity and specificity testing of the ASYLAMP–AuNP assay

To test the analytical sensitivity of the asymmetric LAMP–AuNP (ASYLAMP–AuNP) assay, genomic DNA from a confirmed ToLCV positive sample at ∼80 ng µL^−1^ was used. Ten-fold serial dilutions from 10^−1^ to 10^−12^ in nuclease-free water were prepared and tested with both the ASYLAMP–AuNP assay and conventional PCR, following the protocols in Sections 2.8 and 2.2. The limit of detection (LOD) was the lowest dilution that consistently gave a positive signal in repeated tests. The theoretical copy number of the target DNA corresponding to the experimentally determined limit of detection was calculated to provide a quantitative estimate of assay sensitivity at the molecular level. The number of genome copies of the sample was calculated using the following formula: number of copies = (quantity of DNA) × (6.022 × 10^23^) ÷ (length of DNA template in bp × 1 × 10^9^ × 650).

To assess analytical specificity, the ASYLAMP–AuNP assay was used on confirmed ToLCV-positive samples and on cDNA from other common tomato-infecting viruses, including ToMV, CMV, TSWV, TMV, INSV, and PVY. Each test included positive and negative controls to monitor performance and rule out non-specific amplification or cross-reactivity.

### 2.10 Analytical validation of the LAMP–AuNP assay

Analytical validation of the developed LAMP–AuNP assay was conducted to assess its robustness, reproducibility, batch consistency, and reagent stability in both laboratory and simulated field conditions. Assay performance was evaluated using purified DNA from a commercial extraction kit and crude plant extracts. Purified DNA was used to assess basic performance with minimal interference, while crude extracts were used to test robustness under realistic field conditions, including plant-derived inhibitors such as polysaccharides, polyphenols, cellular debris, and other metabolites.

Validation experiments were performed using three concentration levels: high-positive, moderate-positive, and near the limit of detection (LOD). To ensure consistency across a wide range and under low-template conditions, three concentrations: 0.08, 0.008, and 0.0016 fg µL^−1^ based on the assay's LOD were selected. For purified DNA samples, confirmed-positive DNA was serially diluted in nuclease-free water. For crude extract samples, infected tomato homogenates were diluted into a healthy plant extract matrix. This clarified distinction between purified DNA and crude extracts helps highlight sample types used in the experiment.

To assess intra-assay repeatability, 5 replicate reactions were run in a single experiment under identical conditions. To assess inter-assay reproducibility, three independent experiments were performed on different days, using separately prepared reagents. Batch-to-batch variation was evaluated by preparing independent batches of LAMP master mix, primers, and AuNP conjugates. To test reagent storage stability, assay reagents were stored at −20 °C, 4 °C, and 25 °C for up to one month and tested periodically with both high-positive and near-LOD samples. All validation experiments included the positive, negative, and no-template controls. During validation, assay performance was assessed for amplification consistency, stability of AuNP-based color development, signal reproducibility, maintenance of analytical sensitivity, and absence of false-positive or nonspecific reactions. Altogether, these measures ensured a thorough and cohesive evaluation of assay reliability.

### 2.11 Direct detection of Kenyan ToLCV isolates using probe-functionalized gold nanoparticles

Direct colorimetric detection of target DNA was performed using probe-functionalized AuNPs without prior amplification. Briefly, 5 µL of diluted total DNA was denatured at 95 °C for 5 min to generate single-stranded DNA. Immediately thereafter, 5 µL of probe-functionalized AuNPs was added, and the mixture was incubated at 65 °C for 10 min to facilitate sequence-specific hybridization between the target DNA and AuNP-bound probes. Following hybridization, 5 µL of MgSO_4_ (40 mM final concentration) was added to induce salt-mediated AuNP aggregation. Colorimetric responses were visually recorded within 5 minutes. All assays were performed under identical conditions to ensure reproducibility. The cloned AV1 gene was used as the positive control, whilst healthy plant DNA was used as the negative control and nuclease-free water as the no-template control.

### 2.12 Asymmetric LAMP–AuNP assay evaluation of infected field samples

Crude DNA extracts were prepared using the modified APEG buffer method described by Ndudzo *et al.*^40^ and routinely used to detect Kenyan ToLCV isolates in 79 field samples. Briefly, to test the crude extract, approximately 50 mg of plant leaf tissue was ground in a 1.5 mL microcentrifuge tube and then immersed in 200 µL of modified APEG buffer. The tubes were briefly vortexed and centrifuged, and the supernatant was diluted 5 : 1 with nuclease-free water. The diluted extract was immediately tested using the asymmetric LAMP–AuNP assay as described in Sections 2.4 and 2.8. Furthermore, samples identified as positive by the asymmetric LAMP–AuNP assay were evaluated by real-time PCR, according to the manufacturer's guidelines, with a few minor modifications. Briefly, a 10 µL reaction mix consisting of 5 µL of Luna Universal 2× qPCR master mix (New England Biolabs, UK), 0.5 µL each of 10 mM ToLCVF3 and 10 mM ToLCVB3 primers (adapted from Ndudzo *et al.*^25^), 1 µL of DNA template, and 3 µL nuclease-free water was prepared. The qTower384 (Analytik Jena, Endress + Hauser Company) was used for amplification using the following thermal profile: initial denaturation at 95 °C for 2 minutes, followed by 40 cycles of denaturation at 95 °C for 15 seconds, then annealing and extension at 60 °C for 60 seconds. The *pMG-Amp* plasmid served as the positive control, healthy plant DNA as the negative control, and nuclease-free water as the no template control. All reactions were run in triplicate.

### 2.13 Analytical validation and diagnostic performance analysis

The ASYLAMP–AuNP biosensor's diagnostic performance was evaluated using a binary classification system, comparing it with conventional PCR, which served as the reference standard. Field samples (n-79) were independently tested by conventional PCR and ASYLAMP–AuNP assays. PCR results determined the true infection status, and the AuNP assay gave a simple visual result: red for positive and purplish-grey for negative. A 2 × 2 contingency table was used to determine true positives (TP), false positives (FP), false negatives (FN), and true negatives (TN). Based on these values and using standard definitions, diagnostic sensitivity, diagnostic specificity, positive predictive value (PPV), negative predictive value (NPV), overall diagnostic accuracy, and likelihood ratios were calculated. Ninety-five percent confidence intervals (95% CIs) for proportions were estimated using the Wilson score method,^42^ which is reliable for studies with moderate sample sizes. All analyses were conducted using GraphPad Prism v10.6.1.

Receiver operating characteristic (ROC) analysis was used to compare the binary results of the ASYLAMP–AuNP assay with PCR classification. While ROC analysis is often used for continuous diagnostic outputs, it is also well established and statistically valid for binary tests. For tests with only dichotomous outcomes (positive/negative), ROC analysis yields a single point defined by sensitivity and specificity, and the area under the curve (AUC) remains a useful summary of the test's diagnostic discrimination.^43–46^ The area under the ROC curve (AUC) was calculated analytically rather than graphically, following standard statistical approaches for binary diagnostic tests and aligning with common practices in field diagnostics. The area under the ROC curve (AUC) was computed using DeLong's non-parametric method, as implemented in the *pROC* package for R v4.5.3.

For a binary diagnostic test, ROC analysis yields a single operating point, with AUC equivalent to the average of sensitivity and specificity, and AUC was estimated as:
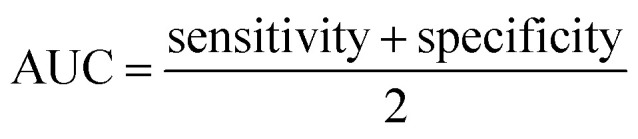


This approach is recommended for qualitative or visually interpreted diagnostics, such as point-of-care, where setting quantitative thresholds is neither practical nor intended.^45,47^ Accordingly, in this study, binary ROC analysis was used to assess the diagnostic performance of the ASYLAMP–AuNP biosensor compared to PCR, since the biosensor is designed for qualitative visual testing in the field, not for quantitative analysis. This approach was chosen because the biosensor's results are qualitative and visually interpreted, and using thresholds from unrelated signals such as *C*_t_ or fluorescence (from the asymmetric LAMP) would not reflect the real-world assay. Binary ROC analysis provides a robust way to summarize sensitivity, specificity, and overall discriminative ability (AUC) without using arbitrary cut-offs that do not reflect real-world use.

The Cohen's kappa (*κ*) statistic was used to measure agreement between the ASYLAMP–AuNP assay and PCR, following standard benchmarks for diagnostic agreement. Agreement was calculated using the formula below:
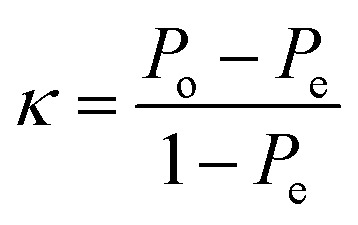
where: *κ* – Cohen's kappa; *P*_o_ – observed agreement; *P*_e_ – expected agreement.

Agreement between ASYLAMP–AuNP biosensor and PCR methods was measured using Cohen's kappa statistic, calculated with the *irr* package in R v4.5.3, which also provides confidence intervals, and significance test estimates.

Analytical validation data were analyzed to assess assay repeatability and reproducibility by examining percent agreement among replicate reactions and independent runs. Because the assay results were qualitative (positive or negative) and the number of replicates was limited, percent agreement was used rather than Cohen's kappa coefficient. This choice was made because Cohen's kappa can yield unstable estimates and unreliable confidence intervals in small samples. Percent agreement was calculated as the proportion of concordant diagnostic results out of all replicate comparisons using the following formula:
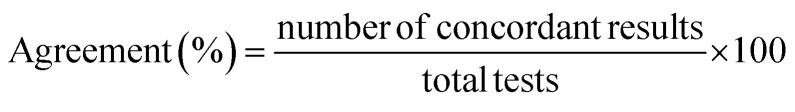


To ensure the approach covered all sample types, separate analyses of purified DNA and crude extract samples were performed at high-positive, moderate-positive, and near-limit-of-detection concentrations. Finally, agreement values near 100% indicated that the assay was highly consistent and reproducible.

## 3 Results and discussion

### 3.1 Detection of Kenyan ToLCV isolates using asymmetric LAMP

In this study, an asymmetric LAMP–AuNP biosensor that uses strand-biased isothermal amplification and plasmonic colorimetric detection was developed for sensitive and specific detection of Kenyan ToLCV isolates. Tests showed that asymmetric LAMP had greater analytical sensitivity, faster amplification, and better performance with AuNP hybridization detection than both conventional PCR and symmetric LAMP.

**Fig. 1:**
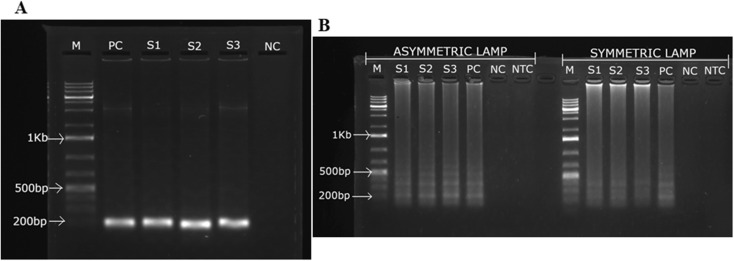
Gel electrophoresis analysis of PCR, symmetric LAMP, and asymmetric LAMP amplification products. (A) Agarose gel electrophoresis of PCR products. (B) Agarose gel electrophoresis of symmetric and asymmetric LAMP amplification products. M-1Kb molecular ladder (O'Gene Ruler 1 Kb Plus DNA Ladder, Thermo Scientific), S1–S3 – positive samples, PC is the positive control, NC is the negative control, and NTC is the no template control.

Initial target amplification was evaluated using conventional PCR, symmetric LAMP, and asymmetric LAMP. Conventional PCR produced a single, clear amplicon of ∼220 bp, matching the expected target size and confirming successful amplification (Fig. 1A).

Both symmetric and asymmetric LAMP reactions showed the typical ladder-like banding pattern on agarose gel electrophoresis, which indicates the formation of concatemeric LAMP amplicons from strand displacement and loop-mediated amplification (Fig. 1B). The banding patterns were similar for both types of LAMP, showing that primer asymmetry did not affect amplification specificity or product quality. Negative and no-template controls showed no bands in either PCR or LAMP, confirming the absence of non-specific amplification or contamination.

### 3.2 Optimization of the asymmetric LAMP assay for detection of Kenyan ToLCV isolates

**Fig. 2:**
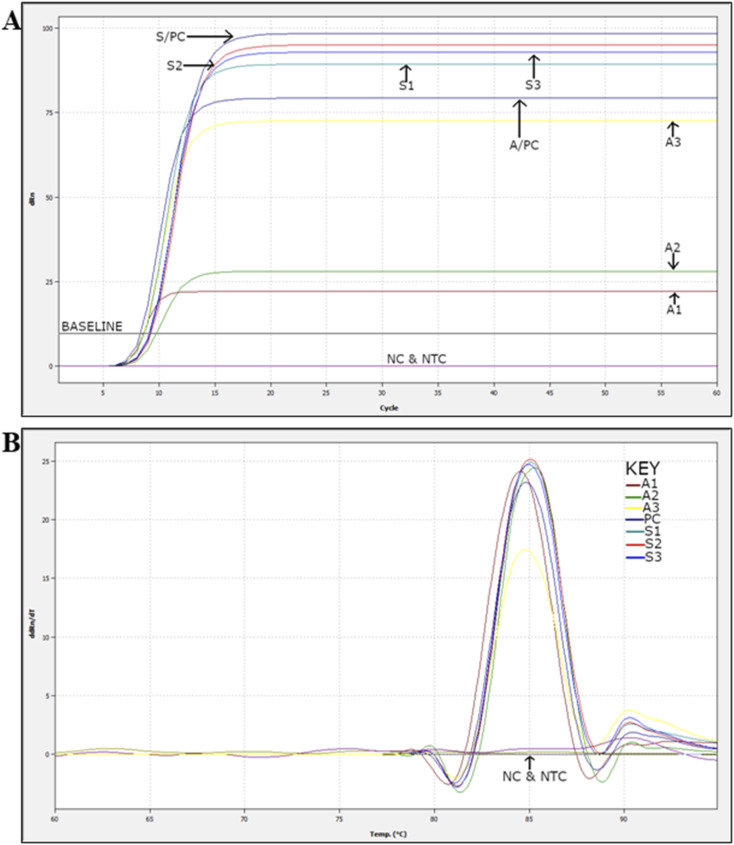
Real-time LAMP analysis of symmetric and asymmetric LAMP. Panel A shows amplification curves for symmetric and asymmetric LAMP products. Panel B presents melt curve analysis for these products. A1–A3 are asymmetric LAMP products, while S1–S3 are symmetric LAMP products. The derivative melt curves (−d*F*/d*T*) show a clear and consistent melting transition at the same temperature in all positive asymmetric LAMP reactions, confirming sequence-specific amplification. The negative control (NC) and no-template control (NTC) did not show any melt peaks.

**Fig. 3:**
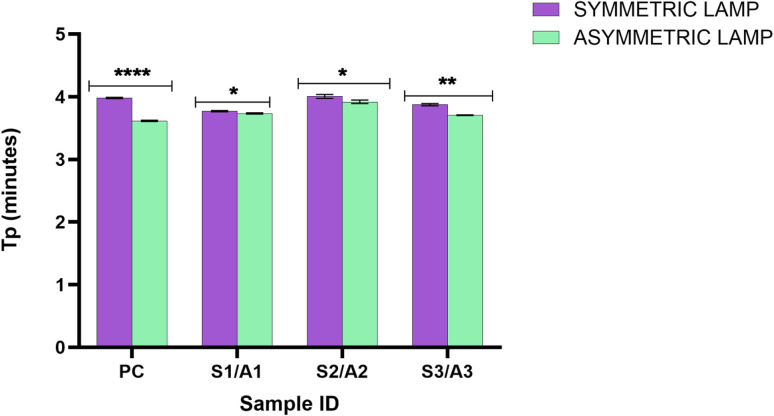
Comparison of time-to-positive (*T*_p_) values between symmetric and asymmetric LAMP reactions. The bar graph displays the mean *T*_p_ values for symmetric (purple) and asymmetric (green) LAMP in the positive control (PC) and representative samples (S1/A1, S2/A2, S3/A3). The values are the mean time-to-positive (minutes) of triplicate readings (mean ± SD; *n* = 3). Bars with * are significantly different according to the unpaired *t*-test (*p* < 0.05). Significance levels are shown as follows: A1/S1 (*P = 0.0110), A2/S2 (*P = 0.0246), A3/S3 (**P = 0.0026), and APC/SPC (****P < 0.0001) and they are considered to be statistically different.

**Fig. 4:**
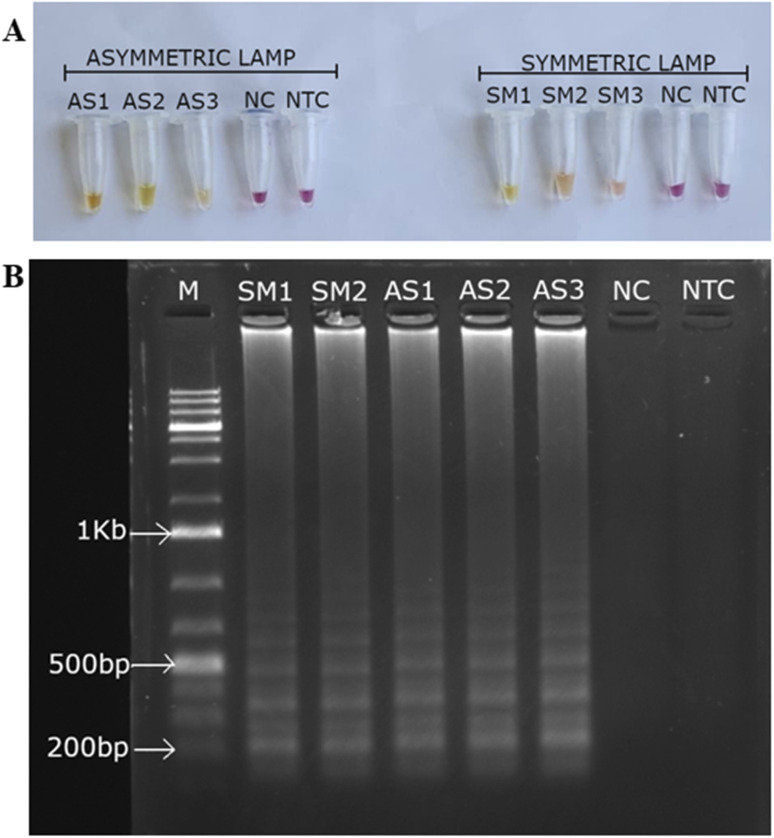
Colorimetric and electrophoretic evaluation of symmetric and asymmetric LAMP. (A) The colorimetric results show symmetric LAMP (SM, FIP : BIP = 1 : 1) and asymmetric LAMP reactions with FIP : BIP ratios of 3 : 1 (AS1), 5 : 1 (AS2), and 8 : 1 (AS3). (B) Agarose gel electrophoresis analysis of symmetric and asymmetric LAMP products at different FIP : BIP ratios. M-1Kb molecular ladder (O'Gene Ruler 1Kb Plus DNA Ladder, Thermo Scientific), NC – negative control and NTC – no-template control.

The performance of symmetric and asymmetric LAMP was compared to assess amplification efficiency, reaction speed, and the product quality for subsequent detection. The asymmetric LAMP assay was designed using established LAMP primer concentration ranges while introducing controlled inner-primer asymmetry to bias strand synthesis. The forward and backward inner primers were set at 2.0 µM and 0.4 µM (FIP : BIP = 5 : 1), which fall within the accepted LAMP inner-primer range and below inhibitory primer loads. An optimization matrix evaluating FIP : BIP ratios of 3 : 1 (A1), 5 : 1 (A2), and 8 : 1 (A3), plus a symmetric control, identified the 5 : 1 ratio as providing the optimal balance between amplification kinetics and strand availability (see Table 1 and Fig. 2–4). This design did not compromise amplification sensitivity or specificity compared to symmetric LAMP.

When tested under the same conditions, asymmetric LAMP showed a much lower cycle threshold (*C*_t_) (SI 2) and lower fluorescence threshold values than symmetric LAMP, indicating that fewer cycles were required for detection (Fig. 2A). This improvement was observed across all replicates and target concentrations, indicating that the performance gains were consistent and not due to random chance.

Melt curve analysis revealed a single, sharp melting peak with consistent *T*_m_ values across all positive samples (Fig. 2B), confirming that using asymmetric primers did not affect specificity. Negative control (NC) and no-template control (NTC) reactions showed no amplification signals in either method confirming sequence-specific amplification and the absence of non-specific products.

Cycle threshold values (SI 2) were used to calculate time-to-positive (*T*_p_) values, which were then compared between symmetric and asymmetric LAMP reactions to assess differences in amplification speed (Fig. 3). For each sample, *T*_p_ values from both reaction types were analyzed using an unpaired, two-tailed *t*-test, with *P* < 0.05 considered statistically significant (SI 3).

Symmetric and asymmetric LAMP reactions were treated as separate experiments because they used different primer compositions and reaction setups, so an unpaired statistical method was used. Each reaction was run three times, and the results are shown as mean ± standard deviation (SD). Asymmetric LAMP had much lower *t*_p_ values than symmetric LAMP in all samples. The positive control comparison (APC *vs.* SPC) showed a very significant difference (*P* < 0.0001, ****). Sample comparisons also showed significant reductions in *t*_p_ for A1/S1 (*P* = 0.0110, *), A2/S2 (*P* = 0.0246, *), and A3/S3 (*P* = 0.0026, **) (SI 3). The results of the unpaired *t*-test showed that *t*_p_ was significantly reduced in all comparisons (*P* < 0.05). The strongest effects were seen in the positive controls (*P* < 0.0001). These results show that amplification kinetics improved while assay reliability remained unchanged.

Agarose gel electrophoresis confirmed the specificity of both amplification formats. Positive reactions showed the expected ladder-like LAMP banding pattern, while NC and NTC reactions showed no detectable bands (Fig. 4B).

Colorimetric detection results supported the fluorescence-based findings. All positive asymmetric LAMP reactions turned yellow, while NC and NTC reactions remained pink (Fig. 4A). The agreement among melt curve analysis, gel electrophoresis, and colorimetric readout confirms the assay's specificity, robustness, and suitability for field use. The results (faster time-to-positivity, maintained melt-curve specificity, and statistically proven performance) show that strand bias can be utilized as a control parameter, not just as a change in primer concentration. The only parameter systematically varied was the FIP : BIP ratio, as the assay was specifically optimized to enhance amplification product output compatible with subsequent AuNP probe hybridization. Excess FIP was used to enrich amplification intermediates with accessible target regions complementary to the AuNP-conjugated probe, prioritizing hybridization efficiency over amplification yield. The key mechanism is that asymmetric primer conditions alter strand-displacement dynamics of LAMP by reducing symmetrical re-priming events; as the limiting primer becomes depleted in later amplification phases, more products with single-stranded or partially exposed regions accumulate. This enhances probe accessibility and improves the efficacy of AuNP-mediated colorimetric detection.

These results concur with previous studies, which reported that using primer asymmetry can improve hybridization efficiency without reducing LAMP sensitivity.^32,48^ Other nucleic acid amplification methods, such as PCR, RPA, and LAMP, have also shown that strand-biased amplification improves probe-based fluorescence, lateral flow results, and both electrochemical and colorimetric biosensors.^30,32,48^ These studies support the use of asymmetric primer designs in LAMP assays to improve hybridization-based detection. Using asymmetric LAMP with AuNPs eliminates the need for a post-LAMP denaturation step at 95 °C to generate single-stranded DNA before hybridization and detection.^49^ Using asymmetric amplification to adjust downstream nanoscale optics is a significant methodological improvement, not just a minor assay refinement.

### 3.3 Analytical sensitivity of asymmetric LAMP

**Fig. 5:**
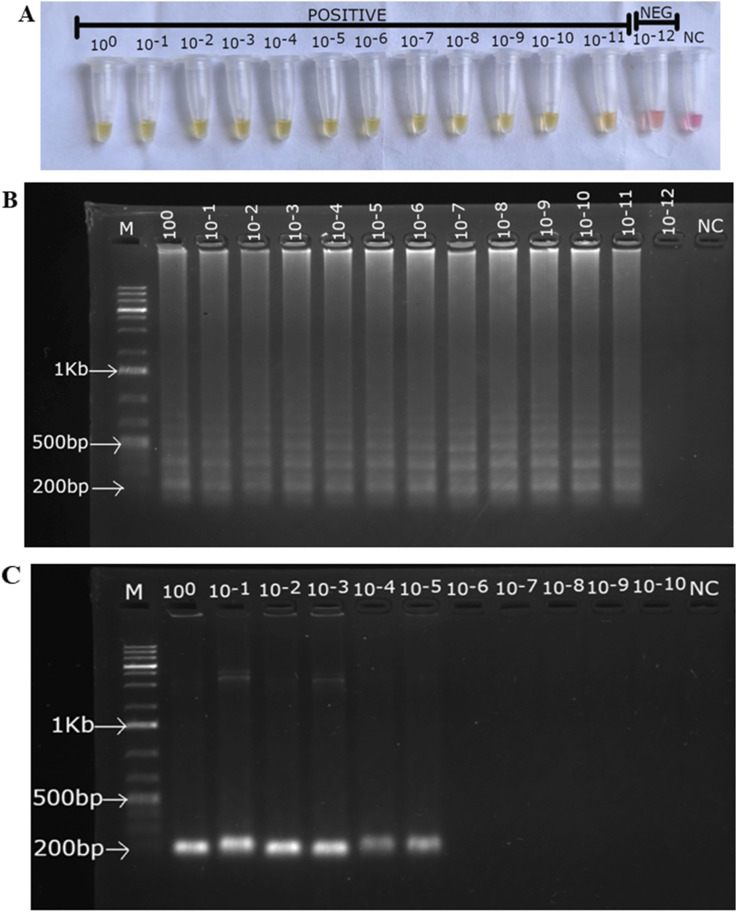
Analytical sensitivity of asymmetric LAMP. (A) Colorimetric detection of asymmetric LAMP products. (B) Agarose gel electrophoresis analysis of asymmetric LAMP products. (C) Agarose gel electrophoresis analysis of conventional PCR products. M-1Kb molecular ladder (O'Gene Ruler 1Kb Plus DNA Ladder, Thermo Scientific), NC-negative control.

The analytical sensitivity of the asymmetric LAMP assay was assessed using serial dilutions of target DNA and compared with conventional PCR as the reference method. Asymmetric LAMP consistently detected target DNA down to a concentration of ∼80 × 10^−11^ ng µL^−1^ (0.0008 fg µL^−1^), which was defined as the limit of detection (LOD) based on reproducible amplification. At this level, positive reactions were easily detected by a color change from pink to yellow (Fig. 5A) and confirmed by agarose gel electrophoresis, which showed the typical ladder-like LAMP banding pattern (Fig. 5B).

Conventional PCR reliably detected the target only down to about 80 × 10^−5^ ng µL^−1^ (800 fg µL^−1^). Below this concentration, no visible amplicon of about 220 bp was seen (Fig. 5C). These results demonstrate that the asymmetric LAMP assay is more sensitive compared to conventional PCR. This result aligns with previous findings on PCR's dependence on thermal cycling, its vulnerability to inhibitors, and its need for exponential amplification of double-stranded DNA.^50^

### 3.4 Analytical specificity of asymmetric LAMP for detection of Kenyan ToLCV isolates

**Fig. 6:**
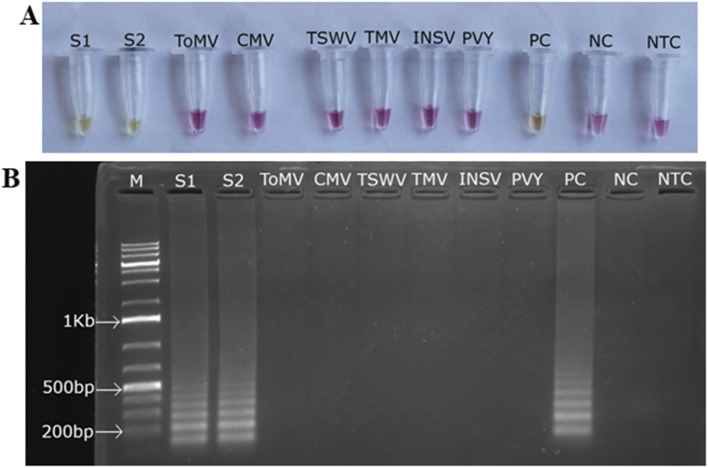
Specificity of the asymmetric LAMP assay for detection of Kenyan ToLCV isolates. (A) Colorimetric detection of asymmetric LAMP products. (B) Agarose gel electrophoresis analysis of asymmetric LAMP products. S1 and S2 – ToLCV positive samples, PC-positive control, NC-negative control, NTC-no-template control, M-1Kb molecular ladder (O'Gene Ruler 1Kb Plus DNA Ladder, Thermo Scientific).

To determine the analytical specificity of the asymmetric LAMP assay, cross-reactivity with several common tomato-infecting viruses was tested. These included ToMV, TMV, CMV, INSV, PVY, and TSWV. Only the ToLCV-positive samples (S1 and S2) (∼80 ng µL^−1^) and the positive control showed amplification, as evidenced by a color change from pink to yellow (Fig. 6A) and confirmed by the ladder-like LAMP banding pattern on agarose gel electrophoresis (Fig. 6B).

There was no amplification or color change in reactions with non-target viruses, the negative control, or the no-template control. This shows that the assay is highly specific and does not cross-react under these test conditions.

### 3.5 Characterization of gold nanoparticles (AuNPs)

**Fig. 7:**
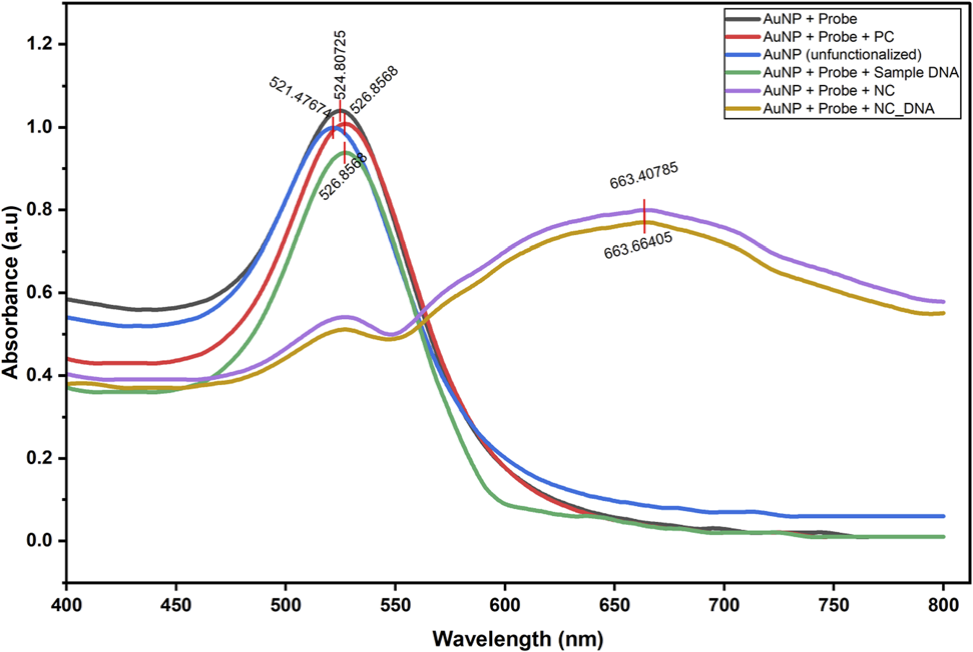
UV-visible spectroscopic characterization of unfunctionalized and functionalized gold nanoparticles for colorimetric detection of Kenyan ToLCV isolates. PC – positive control, NC – negative control, NC_DNA – non-complementary DNA.

Gold nanoparticle conjugates used for colorimetric detection of asymmetric LAMP amplicons were characterized by UV-visible spectrophotometry to validate probe conjugation and hybridization specificity. AuNPs exhibit unique localized surface plasmon resonance properties that make them highly visible, even at low concentrations. This makes them useful for simple, visual diagnostics without complex equipment.^51,52^ Unfunctionalized AuNPs exhibited a distinct surface plasmon resonance (SPR) peak centered at ∼521 nm, signifying well-dispersed nanoparticles. Conjugation of the DNA probe shifted the SPR peak to ∼524 nm, consistent with successful surface modification and a change in the local dielectric environment. Hybridization with complementary asymmetric LAMP-derived DNA resulted in a further shift to ∼527 nm, indicating that the nanoparticles interact with the target in a specific manner (Fig. 7 and SI 4), consistent with stable probe-target interactions and visually retained red coloration.

AuNP conjugates that were incubated with non-complementary targets or negative controls showed broadening of the spectrum and a red-shifted peak at ∼663 nm (Fig. 7), which indicates nanoparticle aggregation and a visible color change. These spectral patterns concur with the analytical specificity results, which show no cross-reactivity with non-target viral templates. The observed spectral and visual changes confirm that the probes were effectively conjugated, that hybridization was sequence-specific, and that the assay can clearly distinguish between complementary and non-complementary target.^53^ In this study, we used UV-Vis spectroscopy to analyze probe-conjugated AuNPs as part of a non-crosslinking nucleic acid biosensing method. In these systems, detection relies on changes in localized surface plasmon resonance (LSPR) that affect colloidal stability and the nanoparticle surface after sequence-specific hybridization, rather than on random nanoparticle aggregation. As a result, UV-Vis spectral analysis is a common method for confirming successful probe attachment and nanoparticle stabilization after hybridization, as changes in absorbance, peak width, and LSPR wavelength indicate events at the nanoparticle surface.^25,52^ The red shift linked to aggregation was observed only in non-complementary and negative control samples, which is consistent with the known plasmon-coupling behavior of AuNPs and shows that complementary targets stabilize the conjugates in a sequence-specific manner.^52^ Recent research shows that using AuNPs with LAMP can lower false positives and improve detection, as seen in viral tests where visual results support rapid isothermal amplification.^54–56^ The main objective of this proof-of-concept study was to validate the functionality of the asymmetric LAMP–AuNP biosensor platform. Earlier research on AuNP-based plasmonic biosensors has shown that UV–Vis spectroscopy provides a rapid and reliable means to assess AuNP functionalization and hybridization in colorimetric nucleic acid detection systems.^51,57^ In this study, we observed consistent UV–Vis spectral shifts, stable colorimetric responses that depended on the target, and negative results in no-template controls. These findings suggest that the signals mainly arose from sequence-specific hybridization rather than nonspecific colloidal aggregation.

### 3.6 Optimization of the ASYLAMP – AuNP assay

**Fig. 8:**
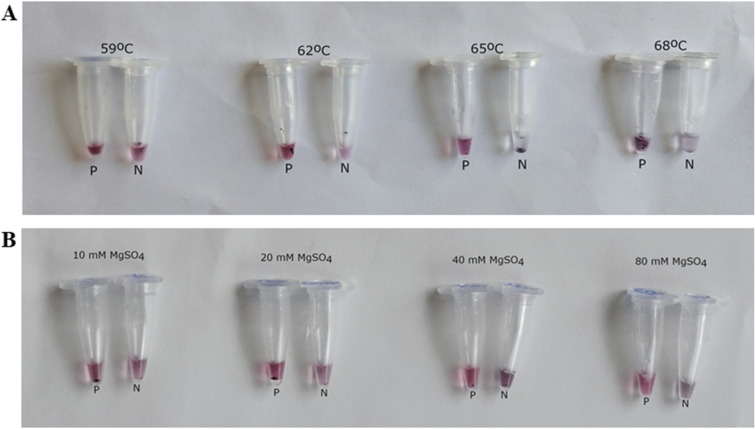
Optimization of asymmetric LAMP–AuNP assay conditions. (A) Shows how different hybridization temperatures (59 °C, 62 °C, 65 °C, and 68 °C) affect assay performance. (B) Shows how varying MgSO_4_ concentrations (10, 20, 40, and 80 mM) influence AuNP-based colorimetric detection after hybridization with asymmetric LAMP products. Positive samples (P) turned red, while negative controls (N) showed salt-induced aggregation, changing color from red to purplish-grey.

To optimize the asymmetric LAMP–AuNP assay, different salt concentrations and amplification temperatures were tested to improve its ability to distinguish positive from negative results. To assess the effect of ionic strength on plasmonic signal development, asymmetric LAMP products were hybridized to probe-functionalized AuNPs in the presence of MgSO_4_ concentrations ranging from 10 to 80 mM. At 40 mM MgSO_4_, positive samples maintained a stable red color, while negative controls turned purplish-blue to blue-grey, showing a clear, consistent difference (Fig. 8B). At this level, the ionic strength best balanced the AuNP surface charges, reduced unwanted aggregation, and improved signal contrast for plasmonic biosensing.^58^ This agrees with earlier findings by Seetang-Nun *et al.*^59^ on the detection of white spot syndrome virus (WSSV).

An optimal temperature of 65 °C was found to be effective and efficient for probe-target binding without disrupting the nanoparticle conjugates (Fig. 8A) as it yielded the strongest colorimetric contrast. Based on this, 40 mM MgSO_4_ and 65 °C were used in all later assays.

### 3.7 Sensitivity and specificity of the ASYLAMP–AuNP biosensor

**Fig. 9:**
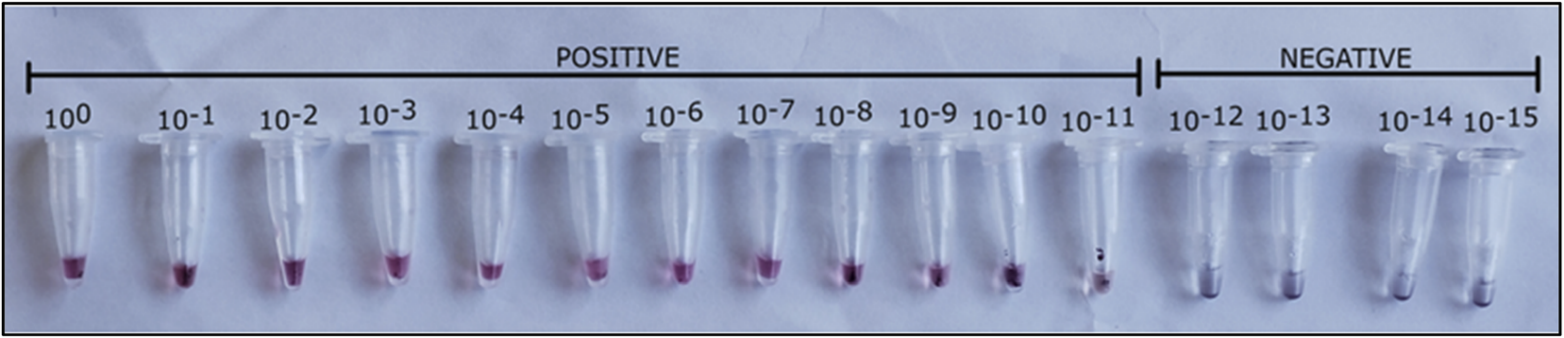
Sensitivity analysis of the asymmetric LAMP–AuNP assay. Colorimetric detection of asymmetric LAMP products after hybridization with probe-functionalized gold nanoparticles using serially diluted target DNA.

The analytical sensitivity of the ASYLAMP–AuNP assay was evaluated using tenfold serial dilutions of ToLCV DNA and compared directly with that of conventional PCR. The assay could reliably detect target DNA at concentrations as low as approximately 80 × 10^−11^ ng µL^−1^ (0.0008 fg µL^−1^). At this level, there was a clear and consistent color change from red (positive reaction) to purplish-grey (negative or below-threshold reaction) (Fig. 9). Under these conditions, the ASYLAMP–AuNP assay maintained the same detection limit as asymmetric LAMP alone, showing that the plasmonic readout did not reduce sensitivity.^25,60^

Samples with concentrations above the limit of detection exhibited the red color, showing that the AuNPs remained stable and dispersed after probe-target hybridization. When the samples were diluted further, the AuNPs aggregated, resulting in a color shift to purplish-grey. These findings show that the asymmetric LAMP–AuNP platform is highly sensitive and can detect low-abundance targets with a simple visual inspection. The conventional colorimetric LAMP assay and the AuNP-coupled LAMP assay shared the same analytical detection threshold (0.0008 fg µL^−1^); however, adding gold nanoparticles enhances assay performance by increasing specificity, signal clarity, and reliability of visual interpretation.^21^ The AuNP-based detection method introduces an additional layer of target confirmation through probe-mediated nanoparticle–nucleic acid binding, strengthening sequence-specific identification. Furthermore, the distinct optical characteristics of AuNPs produce pronounced, readily visible color shifts upon aggregation or dispersion, thereby enabling clear differentiation between positive and negative reactions.

The results of this study are consistent with earlier research showing that combining isothermal amplification with AuNP-based biosensing can enable rapid, accurate detection of plant viruses. Previous work has demonstrated that LAMP achieves high sensitivity in detecting begomoviruses such as tomato leaf curl New Delhi virus (ToLCNDV),^12^ and tomato leaf curl Bengaluru virus.^16^ However, many of these tests relied on techniques such as turbidity checks, fluorescent dyes, or agarose gel electrophoresis, which are less practical in field conditions due to the risk of contamination and their complexity. By integrating AuNP-based biosensing, this study offers a simpler, more field-friendly approach for detecting plant viruses.

AuNP-assisted biosensing is gaining attention for its ease of use and high sensitivity. In this study, the target copy number was calculated to be 3.3 copies per µL. This means the assay can detect about 3 target DNA molecules in each microliter. This shows it has very high analytical sensitivity, nearly capable of detecting single DNA copies. This greater sensitivity probably originates from the asymmetric primer design, which favors the production of single-stranded products. This increases the number of target sequences available for probe binding and helps stabilize the gold nanoparticles. Previous AuNP studies have also reported almost similar sensitivities. For example, Lavanya *et al.*^27^ developed a thiolated AuNP probe assay that could detect begomovirus at very low levels, with a limit of detection of 500 ag µL^−1^. Similarly, Wang and Yang^26^ demonstrated that TYLCV can be detected quickly using an RPA-AuNP platform that does not require complex equipment. In addition, researchers have used comparable AuNP-based methods to detect banana bunchy top virus^61^ and potato virus Y using AuNP-conjugated antibodies for rapid viral detection.^62^

**Fig. 10:**
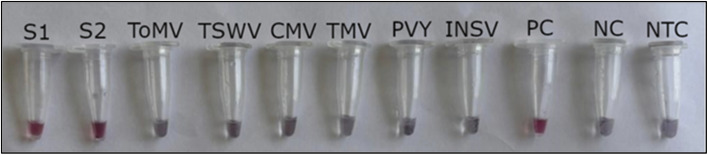
Specificity assessment of the asymmetric LAMP–AuNP assay. Colorimetric discrimination between ToLCV-positive samples and other tomato-infecting viruses using the asymmetric LAMP–AuNP platform.

The analytical specificity of the asymmetric LAMP–AuNP assay was evaluated using ToLCV-positive samples alongside several non-target tomato-infecting viruses. The specificity of the ASYLAMP–AuNP assay depended on the DNA probe being complementary to the DNA target. Thus, the biosensor was tested with 6 types of non-complementary DNA and 2 types of complementary DNA to determine its specificity. The first specificity test was performed by adding the AuNP and the DNA probe solution to non-complementary DNA, and the color change was observed visually. Spectrophotometric analysis was performed to detect any wavelength shift or color change caused by nonspecific nanoparticle aggregation. With non-complementary DNA, the wavelength peak shifted to 663 nm (Fig. 7), and the color changed from pink to purplish-grey (Fig. 10). The second specificity test was performed using both the complementary AuNP-DNA probe and the DNA target. No color change was observed in the biosensor solution, which remained red (Fig. 10). Spectrophotometry showed a slight shift in wavelength from 524 to 527 nm (Fig. 7).

None of the tested non-target viruses showed cross-reactivity, which shows that the assay is highly specific. This selectivity stems from the use of virus-specific primers and AuNP-conjugated probes targeting conserved regions of the coat protein gene, thereby minimizing cross-reactivity even when other microbes are present. Similar systems, such as AuNP-LAMP assays for begomoviruses, including tomato leaf curl New Delhi virus (ToLCNDV), also showed 100% specificity in field samples and did not amplify non-target DNA.^56,58^ The negative and no-template controls also showed color changes associated with aggregation, confirming that the signal appeared only when the target was amplified and hybridized.

### 3.8 Direct functionalized AuNP – genomic DNA assay

**Fig. 11:**
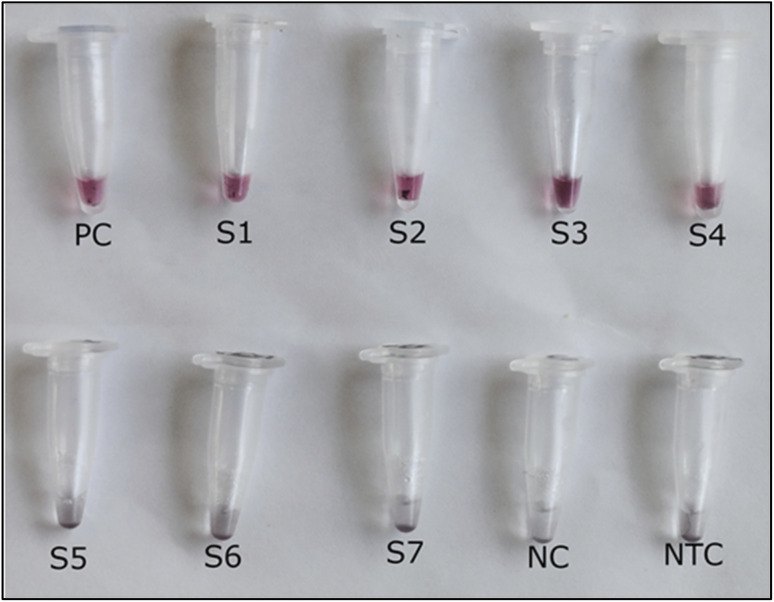
Colorimetric evaluation of the functionalized AuNP direct DNA hybridization assay. Colorimetric response of probe-functionalized gold nanoparticles following direct hybridization with extracted target DNA, without using asymmetric LAMP amplification first.

Direct DNA hybridization assays without amplification were also used to establish a baseline for AuNP-conjugate performance. This direct test served as a rigorous control to check the specificity and functionality of the AuNP conjugates. Positive reactions exhibited the red color. In negative reactions, the color changed from red to purplish blue or grey, indicating that the AuNPs aggregated in the absence of matching target sequences (Fig. 11).

These results confirm that the AuNP probe can discriminate between target and non-target DNA. This step confirms the conjugates' specificity and function and rules out amplification artifacts, such as primer dimers, as a source of bias. Comparative testing like this is standard in AuNP biosensor development and supports amplification-free applications in settings with limited resources. For example, non-amplified AuNP assays for genomic DNA have reached femtomolar limits of detection, supporting their use as controls and alternatives for rapid screening.^63,64^ The main drawback is that genomic DNA must be denatured at 95 °C before hybridization.

### 3.9 Analytical validation results

The asymmetric LAMP–AuNP biosensor demonstrated strong analytical performance, reproducibility, specificity, and storage stability, with high sensitivity even at low template concentrations for ToLCV detection. Results were consistent across intra-assay, inter-assay, and batch tests at higher concentrations, indicating stable amplification chemistry and reliable AuNP-mediated hybridization (SI). Consistency dropped near the detection limit (0.0016 fg µL^−1^), particularly in crude extracts (SI 5). This aligns with earlier findings, which report that stochastic amplification effects and inhibitory phytochemicals affect LAMP performance at extremely low template concentrations.^12^ The increased variability in crude extracts was likely due to plant-derived inhibitors, including polysaccharides, phenolic compounds, and secondary metabolites, which interfere with polymerase activity and nucleic acid hybridization.^27^ Analysis of batch-to-batch variation showed full agreement (100%) at 0.08 fg µL^−1^ for independently prepared reagent batches. However, at 0.0016 fg µL^−1^, agreement dropped to 50%, likely due to small differences in reagent composition and AuNP reaction dynamics near the detection limit (SI 5). Lower consistency at very low template concentrations is likely caused by slight differences in reagent composition, enzyme activity, primer performance, or AuNP aggregation; these effects are more pronounced near the detection limit. Regarding reagent storage stability, assay performance was preserved at −20 °C and 4 °C for 28 days, with 100% agreement at 0.08 fg µL^−1^. Conversely, consistency decreased at 25 °C and at concentrations near the limit of detection after longer storage (SI 5). Decreased sensitivity for low-template samples is likely due to persistent thermal effects on enzyme activity, primer stability, and AuNP reaction dynamics, which are more pronounced near the detection limit. Throughout all validation experiments, positive controls consistently tested positive, while negative and no-template controls remained negative, confirming the assay's specificity and absence of contamination or nonspecific amplification.

### 3.10 Evaluation of field samples using asymmetric LAMP–AuNP biosensor

The applicability of the asymmetric LAMP–AuNP biosensor for field diagnostics was evaluated using 79 leaf samples collected from tomato (*n* = 67) and chili pepper (*n* = 12) plants, including both symptomatic and asymptomatic cases. DNA was extracted with a modified APEG-based crude lysis buffer and then tested with the asymmetric LAMP–AuNP method. Out of the 79 samples, 47 (59.5%) were positive with the biosensor (SI 6), while 44 (55.7%) were positive by conventional PCR (Table 2).

**Table 2:** Comparative detection of field samples using asymmetric LAMP–AuNP, conventional PCR, and real-time qPCR

Sample code	Sampling area	Sampling crop	Symptomatology	Conventional PCR	Asymmetric LAMP	Asymmetric LAMP–AuNP assay	Real-time qPCR
Z1	Embu	Tomato	Symptomatic	+	+	+	+
Z2	Embu	Tomato	Symptomatic	+	+	+	+
Z3	Embu	Chili pepper	Asymptomatic	−	−	−	
Z4	Embu	Tomato	Symptomatic	+	+	+	+
Z5	Embu	Tomato	Symptomatic	−	+	+	+
Z6	Embu	Tomato	Symptomatic	+	+	+	+
Z7	Embu	Chili pepper	Symptomatic	−	−	−	
Z8	Embu	Tomato	Asymptomatic	−	−	−	
Z9	Kirinyaga	Tomato	Symptomatic	−	−	−	
Z10	Kirinyaga	Tomato	Symptomatic	+	+	+	+
Z11	Kirinyaga	Tomato	Symptomatic	+	+	+	+
Z12	Kirinyaga	Tomato	Symptomatic	−	−	−	
Z13	Kirinyaga	Tomato	Symptomatic	+	+	+	+
Z14	Kirinyaga	Tomato	Symptomatic	+	+	+	+
Z15	Kirinyaga	Tomato	Symptomatic	+	+	+	+
Z16	Kirinyaga	Chili pepper	Symptomatic	+	+	+	+
Z17	Kirinyaga	Tomato	Symptomatic	−	−	−	
Z18	Kirinyaga	Tomato	Symptomatic	+	+	+	+
Z19	Kirinyaga	Tomato	Symptomatic	+	+	+	+
Z20	Kirinyaga	Tomato	Asymptomatic	−	−	−	
Z21	Kirinyaga	Chili pepper	Symptomatic	+	+	+	+
Z22	Kirinyaga	Tomato	Symptomatic	+	+	+	+
Z23	Kirinyaga	Tomato	Symptomatic	−	−	−	
Z24	Kirinyaga	Tomato	Symptomatic	+	+	+	+
Z25	Kirinyaga	Tomato	Symptomatic	+	+	+	+
Z26	Kirinyaga	Tomato	Symptomatic	−	−	−	
Z27	Kirinyaga	Tomato	Symptomatic	−	−	−	
Z28	Baringo	Tomato	Symptomatic	+	+	+	+
Z29	Baringo	Tomato	Symptomatic	−	+	+	+
Z30	Baringo	Tomato	Symptomatic	+	+	+	+
Z31	Baringo	Chili pepper	Symptomatic	+	+	+	+
Z32	Baringo	Tomato	Symptomatic	−	−	−	
Z33	Baringo	Tomato	Symptomatic	+	+	+	+
Z34	Baringo	Tomato	Symptomatic	−	−	−	
Z35	Baringo	Tomato	Symptomatic	+	+	+	+
Z36	Baringo	Tomato	Symptomatic	+	+	+	+
Z37	Baringo	Tomato	Symptomatic	−	−	−	
Z38	Narok	Tomato	Symptomatic	+	+	+	+
Z39	Narok	Tomato	Symptomatic	+	+	+	+
Z40	Narok	Chili pepper	Symptomatic	+	+	+	+
Z41	Narok	Tomato	Asymptomatic	−	−	−	
Z42	Narok	Tomato	Symptomatic	+	+	+	+
Z43	Narok	Chili pepper	Symptomatic	+	+	+	+
Z44	Narok	Tomato	Symptomatic	+	+	+	+
Z45	Narok	Tomato	Symptomatic	+	+	+	+
Z46	Narok	Tomato	Symptomatic	−	+	+	+
Z47	Narok	Tomato	Symptomatic	+	+	+	+
Z48	Narok	Chili pepper	Symptomatic	−	−	−	
Z49	Narok	Tomato	Symptomatic	+	+	+	+
Z50	Narok	Tomato	Symptomatic	+	+	+	+
Z51	Laikipia	Tomato	Symptomatic	+	+	+	+
Z52	Laikipia	Tomato	Symptomatic	−	−	−	
Z53	Laikipia	Tomato	Symptomatic	−	−	−	
Z54	Laikipia	Tomato	Symptomatic	+	+	+	+
Z55	Laikipia	Chili pepper	Asymptomatic	−	−	−	
Z56	Laikipia	Chili pepper	Symptomatic	−	−	−	
Z57	Laikipia	Tomato	Symptomatic	+	+	+	+
Z58	Laikipia	Tomato	Symptomatic	+	+	+	+
Z59	Laikipia	Tomato	Symptomatic	−	−	−	
Z60	Laikipia	Chili pepper	Symptomatic	+	+	+	+
Z61	Laikipia	Tomato	Symptomatic	+	+	+	+
Z62	Laikipia	Tomato	Symptomatic	+	+	+	+
Z63	Laikipia	Tomato	Symptomatic	+	+	+	+
Z64	Laikipia	Tomato	Symptomatic	−	−	−	
Z65	Kajiado	Tomato	Symptomatic	+	+	+	+
Z66	Kajiado	Tomato	Symptomatic	−	−	−	
Z67	Kajiado	Tomato	Symptomatic	−	−	−	
Z68	Kajiado	Tomato	Symptomatic	−	−	−	
Z69	Kajiado	Chili pepper	Symptomatic	+	+	+	+
Z70	Kajiado	Tomato	Symptomatic	−	−	−	
Z71	Kajiado	Tomato	Symptomatic	+	+	+	+
Z72	Kajiado	Tomato	Symptomatic	−	−	−	
Z73	Kajiado	Tomato	Symptomatic	−	−	−	
Z74	Kajiado	Tomato	Symptomatic	−	−	−	
Z75	Kajiado	Tomato	Symptomatic	+	+	+	+
Z76	Kajiado	Tomato	Symptomatic	−	−	−	
Z77	Kajiado	Tomato	Asymptomatic	−	−	−	
Z78	Kajiado	Tomato	Symptomatic	−	−	−	
Z79	Kajiado	Tomato	Symptomatic	−	−	−	

This higher detection rate aligns with earlier studies that found LAMP to be more sensitive in samples with low viral loads or high inhibitor levels.^50,65–67^ All samples that tested positive with the asymmetric LAMP–AuNP method were also confirmed by real-time qPCR (SI 7), demonstrating complete agreement with the reference method. None of the asymptomatic samples tested positive with either the asymmetric LAMP–AuNP assay or real-time qPCR, which shows high specificity under field-like extraction conditions. These results show that the asymmetric LAMP–AuNP biosensor is robust and more sensitive for detecting target virus infections in field-collected plant samples using minimally processed DNA.

The system's design, which favors single-stranded products, likely made it easier to detect target regions, helping the biosensor detect low-titer infections that PCR might fail to detect due to its higher detection threshold and sensitivity to inhibitors. No false positives were found in symptom-free samples, demonstrating the biosensor's accuracy in detecting latent infections. This strong field performance suggests it could be instrumental in resource-constrained settings, such as sub-Saharan Africa, where ToLCV outbreaks worsen food insecurity. Similar isothermal AuNP tests for plant viruses have shown 87–100% sensitivity in on-site testing, allowing timely interventions without complex equipment.^23,50,58,68^

### 3.11 Diagnostic accuracy of the ASYLAMP–AuNP biosensor

Of the 79 field samples tested, 44 were positive by both PCR and ASYLAMP–AuNP, showing strong agreement between the two methods. Three samples tested positive with ASYLAMP–AuNP but were negative by PCR, and the biosensor did not miss any PCR-positive samples. All the other samples were negative for both methods. According to the contingency analysis (SI 8b), the ASYLAMP–AuNP biosensor had a diagnostic sensitivity of 100%, meaning it correctly identified all infected samples, and a diagnostic specificity of 91.4%, indicating few false positives (Table 3 and SI 8b). The absence of false negatives is particularly important for plant virus surveillance, where missed infections can contribute to rapid disease spread. Three samples were negative by conventional PCR but positive by the asymmetric LAMP–AuNP assay, and all showed concordant positive results by qualitative real-time PCR. The results indicate that the discrepancy in the reactions may be due to detection of low virus titers below the analytical sensitivity of conventional PCR, rather than to nonspecific amplification. The positive predictive value (PPV) and negative predictive value (NPV) demonstrated that both positive and negative biosensor results were highly reliable in the tested samples. Likelihood ratio analysis showed that a positive test result substantially increased the likelihood of ToLCV infection, whereas a negative result greatly reduced it (Table 3; SI 8a and 8b).

**Table 3:** Analysis of diagnostic parameters

Diagnostic parameter	Value	95% Cl
Sensitivity	1.000	0.9197–1.000
Specificity	0.9143	0.7762–0.9704
Positive predictive value	0.9362	0.8284–0.9781
Negative predictive value	1.000	0.8928–1.000
Diagnostic accuracy	0.962	
Likelihood ratio (positive)	11.67	
Likelihood ratio (negative)	0.0	
Cohen's kappa (agreement analysis)	0.922 (near perfect agreement)	0.703–1.000
ROC AUC	0.957	0.9101–1.000 (DeLong)

Agreement analysis demonstrated substantial to almost-perfect concordance between the ASYLAMP–AuNP biosensor and PCR, with a high Cohen's *κ* value of 0.922. The high *z*-value (8.223) and very low *p*-value (2.220446 × 10^−16^) show that this agreement is statistically significant and unlikely to be due to chance.

**Fig. 12:**
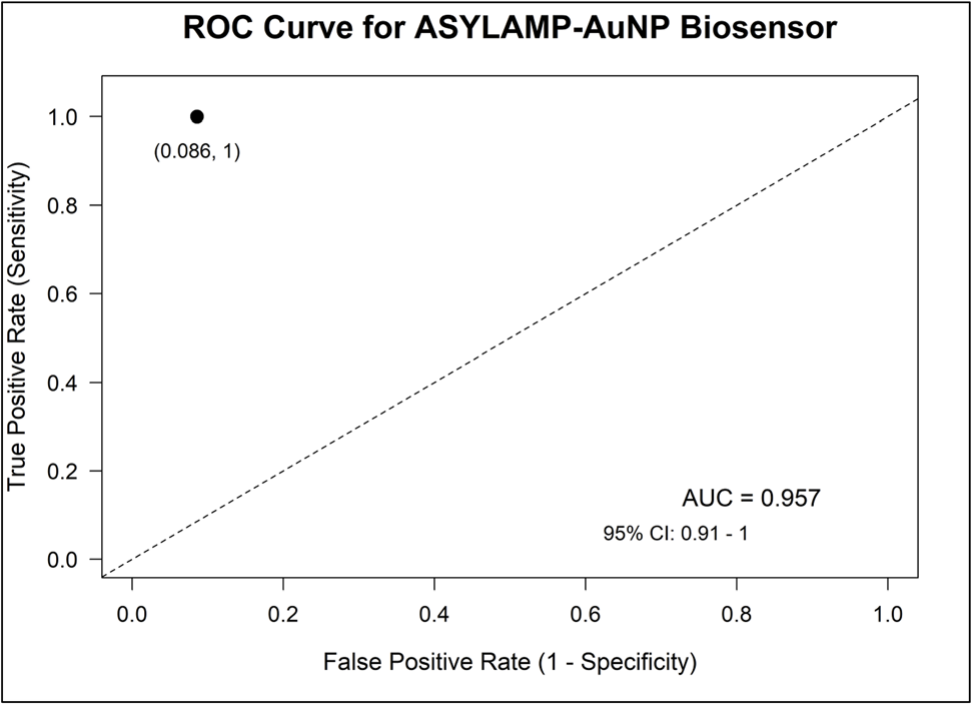
ROC AUC analysis of the ASYLAMP–AuNP biosensor for the detection of Kenyan ToLCV isolates showing a single binary point.

Using binary ROC analysis in this study is consistent with previous research, which shows that ROC-based metrics remain useful for both positive and negative classifiers.^44,45^ In such cases, the ROC curve becomes a single point showing sensitivity and specificity, and the AUC reflects the probability that the test accurately discriminates infected from non-infected samples.^45^ In this study, the binary ROC analysis yielded an AUC of 0.957 (Table 3), with a single binary point demonstrating a false positive rate of 0.086 and a true positive rate of 1.000 (Fig. 12), indicating that the ASYLAMP–AuNP biosensor can clearly distinguish infected from non-infected samples, comparable to PCR.

This approach has been successfully applied in diverse fields, such as medical diagnostics and assay validation, to determine the discriminative potential of binary tests without relying on arbitrary cut-off values.^44,45,69^ The diagnostic parameters derived from binary ROC analysis therefore provide a robust and application-relevant assessment of the ASYLAMP–AuNP biosensor's performance, supporting its suitability for decentralized surveillance and early disease detection.

This proof-of-concept introduces a new diagnostic approach by combining asymmetric LAMP with plasmonic detection, creating a sensitive, specific, and portable tool suitable for low-resource settings. Using asymmetric LAMP with gold nanoparticle colorimetric detection offers key benefits for plant virus testing: fast results in under an hour, easy-to-read results by eye, and minimal equipment requirements. The method is also compatible with crude lysates without sacrificing accuracy, supporting its use in real-world settings and making it valuable for ToLCV monitoring and management. These features reflect broader progress in nanoparticle biosensors, which use the surface plasmon resonance of gold nanoparticles to produce distinct color changes without instruments. This approach has been used for detecting various pathogens in humans, animals, and crops. By adopting asymmetric LAMP as a tool for plasmonic biosensing rather than just another amplification method, this work offers a potential strategy for many nucleic acid diagnostics. Such advances are essential for reducing crop losses, improving disease control, and protecting food security as viral threats increase.

## 4 Conclusion

This study introduces an integrated asymmetric LAMP-gold nanoparticle (AuNP) biosensor for rapid and sensitive detection of Kenyan tomato leaf curl virus isolates. By adjusting the FIP and BIP composition, the LAMP reaction was configured to produce more FIP-derived DNA, thereby improving amplicon binding to the AuNPs. This approach addresses a common challenge in nanoparticle-based nucleic acid detection. The system reached a detection limit of 0.0008 fg µL^−1^, demonstrating high sensitivity, rapidity, and specificity. Tests with field samples using simple DNA extraction reported robust results, with all positive biosensor samples confirmed by real-time qPCR. The key mechanism involves using asymmetric amplification to generate single-stranded DNA, which is then detected through plasmonic interactions between the DNA and the biosensor surface. This creates a scalable, low-equipment diagnostic tool suitable for plant virus monitoring and early disease control in areas with limited resources. This aligns with ongoing efforts to deploy plasmonic LAMP biosensors for crop health monitoring in resource-limited settings. These biosensing strategies make isothermal amplification valuable for field applications, enabling fast pathogen monitoring in agriculture and other fields. Using this platform could improve plant health monitoring and support improved disease management, thereby enhancing food security and agricultural resilience in regions at risk of viral crop diseases. This proof-of-concept asymmetric LAMP–AuNP biosensor lays the foundation for developing and improving highly sensitive nanodiagnostic tools to detect plant viruses. In future work, the focus will be on expanding the analytical and physicochemical characterization of the functionalized nanoparticles using methods such as transmission electron microscopy (TEM), dynamic light scattering (DLS), and surface charge analysis. Broader field validations are also recommended, and the assay should be optimized to further enhance sensitivity, colloidal stability, probe conjugation efficiency, and practical use in point-of-need agricultural diagnostics.

## Author contributions

Conceptualization: Abigarl Ndudzo, Florence Ng'onga, Edith Avedi, Elijah Ateka. Data curation: Abigarl Ndudzo, Florence Ng'onga, Edith Avedi. Formal analysis: Abigarl Ndudzo, Florence Ng'ong'a, Edith Avedi. Funding acquisition: Abigarl Ndudzo. Investigation: Abigarl Ndudzo, Florence Ng'ong'a, Edith Avedi. Methodology: Abigarl Ndudzo, Florence Ng'ong'a, Edith Avedi. Project administration: Abigarl Ndudzo, Florence Ng'ong'a, Edith Avedi, Elijah Ateka. Resources: Abigarl Ndudzo, Florence Ng'ong'a, Edith Avedi. Supervision: Florence Ng'ong'a, Edith Avedi, Elijah Ateka. Software: Abigarl Ndudzo. Validation: Abigarl Ndudzo, Florence Ng'ong'a, Edith Avedi, Elijah Ateka. Writing – original draft: Abigarl Ndudzo. Writing – review and editing: Abigarl Ndudzo, Florence Ng'ong'a, Edith Avedi, Elijah Ateka.

## Conflicts of interest

There are no conflicts to declare.

## Supplementary Material

RA-016-D6RA04196E-s001

## Data Availability

The data supporting this article have been included as part of the supplementary information (SI). Supplementary information: Sl_1: raw gel images; Sl_2: excel file: *C*_t_ values for asymmetric and symmetric LAMP; Sl_3: graphpad file: statistical analysis of asymmetric *vs.* symmetric LAMP data; Sl_4: excel file: UV-Vis spectrophotometry readings; Sl_5: zip file: analytical validation results; Sl_6: results for the colorimetric evaluation of field samples: asymmetric LAMP and asymmetric LAMP-AuNP biosensor assay analysis; Sl_7: real time qPCR amplification and melt curves; Sl_7a: excel file: real time qPCR *C*_t_ values; Sl_8: graphpad file: statistical analysis of diagnostic parameters; Sl_8a: contingency analysis results of diagnostic parameters. See DOI: https://doi.org/10.1039/d6ra04196e.
